# MeCP2 Epigenetic Silencing of *Oprm1* Gene in Primary Sensory Neurons Under Neuropathic Pain Conditions

**DOI:** 10.3389/fnins.2021.743207

**Published:** 2021-11-05

**Authors:** Na Sun, Lina Yu, Yibo Gao, Longfei Ma, Jinxuan Ren, Ying Liu, Dave Schwinn Gao, Chen Xie, Ying Wu, Lieju Wang, Juncong Hong, Min Yan

**Affiliations:** ^1^Department of Anesthesiology, The Second Affiliated Hospital of Zhejiang University School of Medicine, Hangzhou, China; ^2^Department of Anesthesiology, The First People’s Hospital of Huzhou, Huzhou, China; ^3^Department of Anesthesiology, Yuhang First People’s Hospital, Hangzhou, China

**Keywords:** morphine, neuropathic pain, MOR, DNA methylation, histone acetylation

## Abstract

Opioids are the last option for the pharmacological treatment of neuropathic pain, but their antinociceptive effects are limited. Decreased mu opioid receptor (MOR) expression in the peripheral nervous system may contribute to this. Here, we showed that nerve injury induced hypermethylation of the *Oprm1* gene promoter and an increased expression of methyl-CpG binding protein 2 (MeCP2) in injured dorsal root ganglion (DRG). The downregulation of MOR in the DRG is closely related to the augmentation of MeCP2, an epigenetic repressor, which could recruit HDAC1 and bind to the methylated regions of the *Oprm1* gene promoter. MeCP2 knockdown restored the expression of MOR in injured DRG and enhanced the analgesic effect of morphine, while the mimicking of this increase *via* the intrathecal infusion of viral vector-mediated MeCP2 was sufficient to reduce MOR in the DRG. Moreover, HDAC1 inhibition with suberoylanilide hydroxamic acid, an HDAC inhibitor, also prevented MOR reduction in the DRG of neuropathic pain mice, contributing to the augmentation of morphine analgesia effects. Mechanistically, upregulated MeCP2 promotes the binding of a high level of HDCA1 to hypermethylated regions of the *Oprm1* gene promoter, reduces the acetylation of histone H3 (acH3) levels of the *Oprm1* gene promoter, and attenuates *Oprm1* transcription in injured DRG. Thus, upregulated MeCP2 and HDAC1 in *Oprm1* gene promoter sites, negatively regulates MOR expression in injured DRG, mitigating the analgesic effect of the opioids. Targeting MeCP2/HDAC1 may thus provide a new solution for improving the therapeutic effect of opioids in a clinical setting.

## Introduction

Neuropathic pain, affecting approximately 6.9–10% of the global world population, is a major source of suffering and disability, leading to a substantial healthcare costs and lost productivity (Cooper et al., 2017). Neuropathic pain can be categorized into 2 main subgroups: (1) chronic pain with neuropathic characteristics and (2) neuropathic pain associated with a specific condition, including postherpetic neuralgia, trigeminal neuralgia, painful diabetic peripheral neuropathy, and glossopharyngeal neuralgia (van Hecke et al., 2014). Despite their risks, opioids, such as morphine, are the main treatment option for neuropathic pain, but have limited effectiveness in patients with this debilitating disorder (Fields, 2004; Cooper et al., 2017; Els et al., 2017; Martinez-Navarro et al., 2018). This lower efficacy may be due to mu opioid receptor (MOR) downregulation in pain pathways, such as dorsal root ganglion (DRG) and spinal cord, after a lesion or disease of the somatosensory nervous system (e.g., an episode of acute herpes zoster, traumatic nerve injury or neuropathy in diabetes) (Zhang et al., 1998; Takasaki et al., 2006; Lee et al., 2011; Mo et al., 2018; Deshpande et al., 2021). In this study, we selected a model of chronic constriction injury, involving a peripheral mononeuropathy that produces disorders of pain sensation in rodents like those seen in humans (Bennett and Xie, 1988), in order to identify a pivotal mechanism of MOR downregulation in neuropathic pain.

Epigenetic modifications, including DNA methylation, histone acetylation, and long noncoding RNA, are associated with the altered expression of genes in neuropathic pain (Uchida et al., 2010; Li et al., 2019b; Wu et al., 2019). Recently, progress has been made in clarifying the epigenetic mechanisms in the MOR downregulation of neuropathic mice (Liang et al., 2016; Sun et al., 2017; Mo et al., 2018). DNA methylation, like CpG island methylation in gene promoter sites, usually suppresses gene transcription (Razin and Riggs, 1980). The methylated promoter could recruit methyl-CpG-binding domain (MBD) protein, including MBD1–4 and MeCP2 (Lewis et al., 1992; Nan et al., 1998). The MBD family of proteins recruits transcriptional corepressors, such as histone deacetylases (HDACs), to the promoter region of a targeted gene for its silencing (Razin and Riggs, 1980; Nakao, 2001). Thus, it’s likely that the MBD family of proteins is involved in DNA methylation-triggered repression of gene transcription. MeCP2 functions throughout the development of the human brain, and its mutation can lead to several types of cancer and neurodevelopmental diseases (Konduri et al., 2003; Murphy et al., 2011). Its mutation forms the genetic basis of Rett syndrome, a neurological disorder, with the clinical symptom of an altered pain threshold (Downs et al., 2010). In addition, a previous study found that nerve injury induced higher levels of MeCP2 protein in injured DRGs, contributing to the activation of DNA methyltransferase 1 (DNMT1) and VEGFA expression (Manners et al., 2016). MeCP2 is more flexible in combination with methylated CpG sites, whereas other MBD proteins bind to DNA containing at least 12 symmetrical methylated CpGs (Nan et al., 1998). Thus, we hypothesize that MeCP2 may play an essential role in the suppression of *Oprm1* gene transcription.

In recent years, within the field of epigenetics, HDACs have attracted substantial attention in pain modulation. HDACs remove acetyl groups from histones, leading to chromatin compaction, blockade of transcription factors access, and suppression of gene transcription. Prior work showed that HDACs were involved in the regulation of MOR since HDAC inhibitors, such as trichostatin A and valproic acid, which were found to block nerve injury-induced MOR downregulation in the DRG (Uchida et al., 2015). Nonetheless, it is not known which HDAC(s) is involved, and the underlying mechanism has remained unclear. In the present study, we revealed that the hypermethylated *Oprm1* gene promoter could recruit MeCP2 as well as HDAC1 after nerve injury, decreasing acetylation of the *Oprm1* gene promoter region, resulting in the decreased expression of MOR in the DRG.

## Experimental Procedures

### Experimental Animals

**FIGURE 1:**
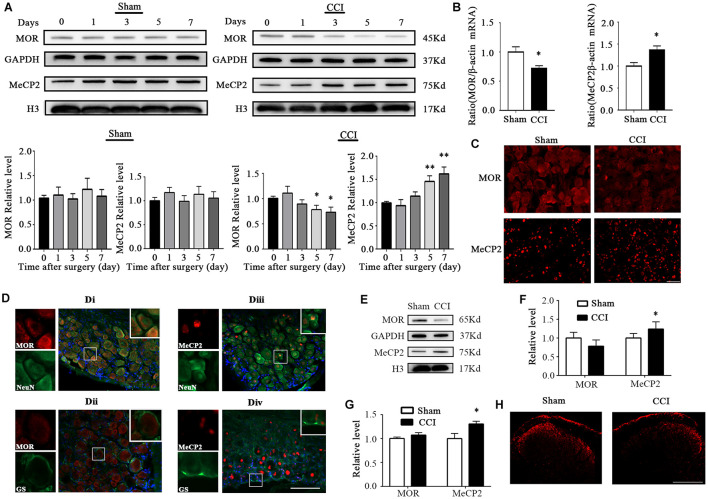
MOR expression was decreased and MeCP2 was increased 7 days after CCI surgery in the injured DRG. **(A)** MOR and MeCP2 protein expression in the ipsilateral L3–5 DRG 7 days after CCI or sham surgery. *n* = 6 biological repeats (18 mice)/time point/group. One-way ANOVA followed by *post hoc* Tukey test. F_group_ (4, 25) = 1.621 for MOR in the sham group, F_group_ (4, 25) = 2.544 for MeCP2 in the sham group, F_group_ (4, 25) = 2.728 for MOR in the CCI group, F_group_ (4, 25) = 6.967 for MeCP2 in the CCI group. *p < 0.05, **p < 0.01, versus day 0 in the CCI group. **(B)** MOR and MeCP2 mRNA expression in ipsilateral DRG 7 days after surgery. *n* = 4 biological repeats (8 mice)/group. *p < 0.05, versus the sham group by two-tailed unpaired Student’s *t*-test. **(C)** Representative images of MOR immunofluorescence in the injured DRG. MOR immunoreactivity was lower on the ipsilateral injured side of the DRG, compared with the level in sham DRG 7 days after CCI. Scale bar: 100 μm. *n* = 3 mice/group. **(D)** Neurons/gliocytes labeled by MOR/MeCP2 and NeuN/GS in the ipsilateral DRG of naïve mice. Nuclei were stained with DAPI. Scale bar: 100 μm. *n* = 3 mice/group. **(E)** MOR and MeCP2 protein levels in L3–5 spinal cord of the injured side 7 days after surgery. *n* = 6 biological repeats (6 mice)/group. **(F)** Quantification of MOR and MeCP2 protein expression of panel **(E)**. *p < 0.05, versus the corresponding sham group by two-tailed unpaired Student’s *t*-test. **(G)** MOR and MeCP2 mRNA levels in L3–5 spinal cord on the injured side 7 days after surgery. *n* = 4 biological repeats (4 mice)/group. *p < 0.05, versus the corresponding sham group by two-tailed unpaired Student’s *t*-test. **(H)** Immunofluorescence staining of MOR in the spinal dorsal horn from sham or CCI mice 7 days after surgery. Scale bar: 200 μm. *n* = 3 mice/group.

**FIGURE 2:**
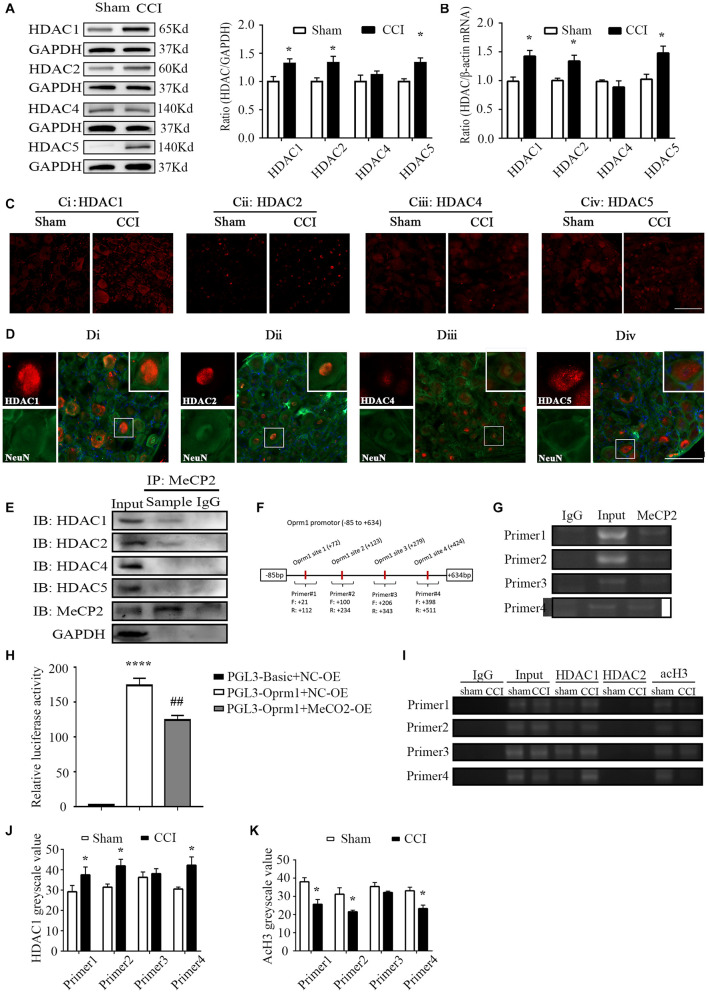
HDAC1 was involved in MOR regulation. **(A)** Protein levels of HDAC1, –2, –4, and 5 in the ipsilateral L3–5 DRG on day 7 from CCI and sham mice. *n* = 6 biological repeats (18 mice)/group. *p < 0.05, versus the corresponding sham group by two-tailed unpaired Student’s *t*-test. **(B)** RNA levels of HDAC1, –2, –4, and 5 in the ipsilateral L3–5 DRG on day 7 from CCI and sham mice. *n* = 4 biological repeats (8 mice)/group. *p < 0.05, versus the corresponding sham group by two-tailed unpaired Student’s *t*-test. **(C)** Representative images of HDAC1/2/4/5 in the ipsilateral DRG on day 7 from CCI and sham mice. HDAC1, –2 and 5 immunoreactivities were increased in the ipsilateral DRG compared with the levels in sham DRG 7 days after CCI. Scale bar: 100 μm. *n* = 3 mice/group. **(D)** Neurons labeled by HDAC1/2/4/5 and NeuN in the DRG of naïve mice. Scale bar: 100 μm. *n* = 3 mice/group. **(E)** Co-IP showed that MeCP2 could bind to HDAC1 and HDAC2 rather than HDAC4 or HDAC5 in the DRG of naive mice. *n* = 20 mice. **(F)** Primer design in the *Oprm1* gene promoter region (–85 to 634) in the following ChIP assays. **(G)** ChIP showed that MeCP2 could bind with the *Oprm1* gene promoter in the four primer regions. Input: collected supernatant. IgG: purified rabbit IgG. **(H)** Luciferase activities were measured at 48 h after transfection of MeCP2-OE or the corresponding negative control (OE-NC) with pGL3-Oprm1 into HEK293 cells. The Renilla plasmid was used as an internal control for the normalization of transfection efficiency. *n* = 3 biological repeats. One-way ANOVA followed by *post hoc* Tukey test. F_group_ (2, 6) = 150.4. ****p < 0.0001, versus PGL-Basic plus NC-OE group, ^##^*p* < 0.01, versus PGL-Oprm1 plus NC-OE group. *Oprm1* gene promoter fragment immunoprecipitated by HDAC1, HDAC2 and acH3 in the ipsilateral DRGs on day 7 after CCI or sham surgery. Input: collected supernatant. IgG: purified rabbit IgG. **(I–K)** Statistical summary of RT–PCR analysis on the binding activity of HDAC1 and acH3. *n* = 4 biological repeats (40 mice)/group. *p < 0.05, versus the corresponding sham group by two-tailed unpaired Student’s *t*-test.

All experimental protocols were approved by the Animal Care and Use Committee of Zhejiang University (Hangzhou, Zhejiang Province, China) and were in accordance with the Declaration of the National Institutes of Health Guide for the Care and Use of Laboratory Animals (Publication No. 80-23, revised 1996). Eight- to twelve-week-old male C57BL/6J mice, weighing about 20 g, provided by Slac Laboratory Animal Co. (Cat No: 5807319, Research Resource Identifier (RRID):MGI: 5807391, Shanghai, China), were used in our experiments. The mice were housed in groups with a 12 h dark/light cycle at an appropriate temperature and humidity in specific pathogen-free cages (5 mice/cage). They had free access to food and water. After the animals had been acclimated in a quiet, temperature-controlled room for 1 h, behavioral studies were performed by other investigators, who were blinded to the drugs used. All behavioral studies were performed between 7 am and 7 pm. The study was not preregistered, and experimental animals were selected at random in this study. For the behavioral tests, there were 12 mice per group. For western blots, there were 6 samples per group, and each DRG sample came from 3 mice, while each spinal cord sample came from 1 mouse. For immunofluorescence, there were 3 mice per group. For quantitative real-time reverse transcription (RT)–PCR, there were 4 samples per group, and each DRG sample came from 2 mice, while each spinal cord sample came from 1 mouse. For pyrosequencing, there were 3 samples per group and each DRG sample came from 6 mice, while each spinal cord sample came from 3 mice. For chromatin immunoprecipitation (ChIP), there were 3 samples per group and each sample came from 10 mice. In addition, samples from 20 mice were used for co-immunoprecipitation (co-IP) experiments. In total, 7 mice died from anesthetic overdose, and 5 mice were excluded based on the exclusion criteria (e.g., normal activity, body weight, gait, and free of wound infection) during the experiment. The precise numbers of mice used are described in the figure legends. To minimize the number of mice used, the experiments for Figures 1, 2 shared the same samples for western blotting and qRT-PCR. All mice were ear tagged with unique numerical identifiers so that blinded experiments could be performed. The randomization function of Microsoft Excel 2013 was used to generate random numbers between 0 and 1 for each mouse. These random numbers were then sorted in ascending order, generating a list that categorized the mice into corresponding groups. Following all procedures, the mice were euthanized by an overdose of isoflurane followed by rapid decapitation with sharp scissors. The experimenter was unaware of each animal’s group membership during experiment.

### Chronic Constrictive Injury Model

In accordance with a previous study (Bennett and Xie, 1988), after sodium pentobarbital (40 mg/kg, intraperitoneal injection; Sigma–Aldrich, St Louis, MI, United States; Cat No: 76-74-4) anesthesia, mice were cut with a small incision beneath the mid-thigh to expose the left sciatic nerve. A unilateral constriction injury was produced with three loose ligatures (6-0 silk thread, spaced at 1-mm intervals) just proximal to the trifurcation. In the sham group, prior steps were identical to the CCI group except that the nerve remained unligated. Finally, both groups had the incised skin closed off with suture. Only mice without any abnormality (e.g., normal activity, body weight, gait, and free of wound infection) were included in this study.

### Drugs and Administration

Morphine (Sigma; Cat No: 25-27-2) and 5-aza-2′-deoxycytidine (5-aza-dC; Sigma; Cat No: 2353-33-5) were dissolved in saline; saline was set as the control. Suberoylanilide hydroxamic acid (SAHA; Selleck Chemicals, Houston, TX, United States; Cat No: S1047) was dissolved in 5% dimethyl sulfoxide (DMSO; Sigma; Cat No: D2650-100 mL); 5% DMSO was set as the control. Morphine was subcutaneously given with a dose of 1.5 mg per kg of body weight. The use of 5-aza-dC was continued for three consecutive days at 5 μg/5 μL daily, 30 min before surgery. SAHA was continued for 7 consecutive days, at a dose of 50 mg/kg in a volume of 5 μL, 30 min before surgery. All drug doses are based on preliminary experiments (Zhou et al., 2014; Cao et al., 2015; Garriga et al., 2018), and did not alter the nociceptive response *per se*. Intrathecal injections were performed manually between L5 and L6 spinal segments, through the theca of the spinal cord into the subarachnoid space (Hylden and Wilcox, 1980). On entry of the needle, a quick flicking motion of the mouse’s tail or leg was noticed. For anesthesia, sodium pentobarbital and isoflurane (Sigma; Cat No: 26675-46-7) were used.

### Assessment of Pain Behavior

#### Thermal Hyperalgesia of the Paw

Thermal hyperalgesia was evaluated by applying the Hargreaves method as described previously (Hargreaves et al., 1988). Briefly, after animals had been acclimated to a special chamber (acrylic 7 cm × 9 cm × 11 cm) for 1 h, a heat source was focused on the posterior hind paw of each mouse before (basal threshold) and after drug or vehicle administration. After each mouse lifted or licked the hind paw, the radiant heat test was stopped. Paw withdrawal latency (PWL) was defined as the time from when the heat source was applied to the endpoint. The radiant heat intensity was set as 60 J, and the cut-off time was set at 20 s. The response latency was recorded as predefined. PWLs were usually averaged from 5 tests.

### Morphine-Induced Analgesia

As described by Yuan et al. (2019), morphine was injected at a predefined time point subcutaneously. Heat tests were conducted before surgery (baseline latency) and 30 min after morphine injection (response latency). The percentage of the maximal possible analgesic effect (% MPAE) = ([response latency − baseline latency]/[cut-off time − baseline latency]) × 100%.

### Immunofluorescence

After sodium pentobarbital anesthesia was induced, we transcardially perfused the mice with 0.9% saline and then with 4% paraformaldehyde. After their removal, the ipsilateral DRGs (L3–L5) were post-fixed in 4% paraformaldehyde for 2 h, and then cryoprotected at 4°C in 30% sucrose until they sank to the bottom of the tube. Sample sections (16 μm) were cut using a cryostat. Next, the sections were incubated in phosphate buffered saline (PBS) containing 5% normal goat serum and 0.3% Triton X-100 at room temperature for 1 h. The sections were then incubated overnight with primary antibody at 4°C. The following primary antibodies were used in this study: mouse anti-NeuN (1:200; Abcam, Cambridge, United Kingdom; Cat No: ab104224, RRID:AB_10711040), mouse anti-GS (1:500; Millipore, Cat No: MAB302, RRID:AB_2110656), rabbit anti-MOR (1:200; Abcam; Cat No: ab10275, RRID:AB_2156356), rabbit anti-MeCP2 (1:200; #3456; Cell Signaling Technology, Danvers, MA, United States; Cat No: 1205-MeCP2, RRID:AB_2632401), rabbit anti-HDAC1 (1:200; Abcam; Cat No: ab19845, RRID:AB_470299), rabbit anti-HDAC2 (1:200; Cell Signaling Technology; Cat No: #2540, RRID:AB_2116822), rabbit anti-HDAC4 (1:200; Cell Signaling Technology; Cat No: #2072, RRID:AB_2232915), and rabbit anti-HDAC5 (1:200; Cell Signaling Technology; Cat No: #20458, RRID:AB_2713973). The sections were then incubated with the appropriate secondary antibody. The secondary antibodies used in the present study included Alexa Fluor^TM^ 488 donkey anti-mouse IgG (H+L) (1:500; Invitrogen, Carlsbad, CA, United States; Cat No: A21202, RRID:AB_141607) and Alexa Fluor^TM^ 594 donkey anti-rabbit IgG (H+L) (1:500; A21207; Invitrogen; Cat No: A21207, RRID:AB_141637). 40, 6-diamidino-2-phenylindole (DAPI) (Abcam, Cat No: ab104139, LOT: GR3281289-1). The sections were examined in a blinded manner using a fluorescence microscope.

### Western Blotting

Western blotting was performed as we detailed previously (Zhou et al., 2014). First, after conducting sevoflurane anesthesia, DRGs (L3–5) (from 3 mice) or 1 spinal cord (the injured side of L3–L5 segments) were pooled together to yield a sufficient protein concentration and then homogenized in lysis buffer (100 μL/sample) containing phenylmethanesulfonyl fluoride (PMSF; Beyotime, Shanghai, China; Cat No: ST506-2). After resting on ice for 30 min, the samples were centrifuged at 13,000 rpm for 10 min at 4°C. We then measured total protein concentrations in supernatants using an Enhanced BCA Protein Assay Kit (Beyotime, Cat No: P0009). Equal amounts of protein (∼60 μg) were loaded into each well for separation by sodium dodecyl sulfate-polyacrylamide gel electrophoresis (SDS-PAGE; 10% gels; Genshare Biology, Shanxi, China; Cat No: JC-PE022). After electrophoresis, proteins were transferred to polyvinylidene fluoride membranes (Millipore, Burlington, MA, United States; Cat No: IPVH00010). We then blocked membranes with nonfat dry milk for 1 h at room temperature, followed by overnight incubation at 4°C with the following primary antibodies: rabbit anti-MOR (1:1000, Abcam, Cat No: ab10275, RRID:AB_2156356), rabbit anti-MeCP2 (1:1000, #3456, Cell Signaling Technology, Cat No: #3456, RRID:AB_2632401), rabbit anti–acetyl-histone H3 (1:1000, Cell Signaling Technology, Cat No: #9649, RRID:AB_823528); rabbit anti-histone H3 (1:1000, Cell Signaling Technology, Cat No: #9715, RRID:AB_331563); rabbit anti-HDAC1 (1:200, Abcam, Cat No: ab19845, RRID:AB_470299), rabbit anti-HDAC2 (1:200, Cell Signaling Technology, Cat No: #2540, RRID:AB_2116822), rabbit anti-HDAC4 (1:200, Cell Signaling Technology, Cat No: #2072, RRID:AB_2232915), rabbit anti-HDAC5 (1:200, Cell Signaling Technology, Cat No: #20458, RRID:AB_2713973); and mouse anti-GAPDH (1:1000, Beyotime, Cat No: AF0006). The membranes were then incubated with horseradish peroxidase-conjugated secondary antibody for 1 h at room temperature. The acquired blots were analyzed accordingly. Membranes were exposed to X-ray film, and band densities were calculated using ImageJ 2.0.

### Quantitative Real-Time–PCR Analysis

After sevoflurane anesthesia, DRGs from 2 mice or 1 spinal cord were pooled together. We concentrated total RNA with TRIzol reagent (Invitrogen, Cat No: 15596-026) and synthesized cDNA with a Thermo Scientific Verso cDNA synthesis kit (ABgene; Thermo Scientific, Waltham, MA, United States; Cat No: RR036A). We performed qRT–PCR with a DyNAmo Flash SYBR Green qPCR kit (Thermo Scientific, Cat No: RR820). Thermal cycle conditions were set as the following: 95°C for 3 min; 40 cycles of 95°C for 15 s, 58°C for 30 s, 72°C for 30 s; and 72°C for 30 min. A StepOnePlus real-time PCR system (Thermo Fisher Scientific, Cat No: 2725213) was used for reactions. β-actin was used as a house-keeping gene for data normalization. For all primers, please refer to Supplementary Table 1.

### Pyrosequencing

After sevoflurane anesthesia, L3–L5 DRGs were harvested 7 days after CCI or sham surgery. DRGs from 6 mice and spinal cord from 3 mice, were separately pooled for DNA extraction (Qiagen, Hilden, Germany; Cat No: 69506). Methylation treatment of genomic DNA was carried out using a QiagenEpiTect Bisulfite Kit (Qiagen, Cat No: 59104) in accordance with the manufacturer’s instructions. A region of the *Oprm1* gene promoter from –85 to +634 bp, which consists of 17 CpG sites, was divided into eight regions and amplified. For the pyrosequencing assay, after amplifying the bisulfite DNA with eight pairs of primers of the *Oprm1* gene (Supplementary Table 2), binding buffer, streptavidin-sepharose beads, and PCR products were prepared for the binding reaction. The plate was then placed on a shaker for at least 15 min, but no longer than 1 h. Pyrosequencing was then performed. For the clone-sequencing assay, the same promoter fragment was amplified. PCR products were transformed into competent cells. Finally, PCR amplification and direct sequencing were carried on after an overnight bacterial culture (Taylor et al., 2007; Tost and Gut, 2007).

### siRNA Preparation

For siRNA injection, MeCP2 siRNA and its negative control (NC) siRNA were purchased from Genomeditech company. The three sequences were dissolved using Entranster^TM^ *in vivo* transfection reagents (Engreen Biosystem Co., Ltd., Auckland, New Zealand; Cat No: 18668-11-1) at a volume of 10 μL (100 pmol in total) for four continuous days intrathecally 30 min before surgery. *In vivo* transfection reagents served as a vehicle. 7 days after surgery, DRGs were collected. The siRNAs of MeCP2 and related primer duplexes are listed in Supplementary Table 3.

### Virus Preparation

Recombinant adeno-associated virus 5 (AAV5)–MeCP2 [(Cat No: pSE3556, titer: 3.23 × 10^13^ VG/mL) (VG/mL means viral genome copy number per ml of virus solution)] and negative control AAV5-enhanced green fluorescence protein (EGFP; Cat No: Pmt423, Titer: 2.52 × 10^13^ VG/mL) were supplied by Shanghai SunBio Medical Biotechnology Co., Ltd. (Shanghai, China) and infused intrathecally (5 × 10^10^ VG in total) through the theca of the L5 and L6 segments of the spinal cord into the subarachnoid space. As previously reported (Lisowski et al., 2014), 30 days were needed to highly express full-length MeCP2 in the DRGs of mice.

### Chromatin Immunoprecipitation Assay

Following the manufacturer’s instructions (EZ-Magna ChIP^TM^ A/G; Cat No: #17-10086) and a previous study (Huang et al., 2019), we performed ChIP assays. A homogenate of the injured side of L3–L5 DRGs of 10 mice was cross-linked with 1% formaldehyde, and glycine was then added to quench the reaction. After centrifugation and resuspension, the collected pellet was sonicated with eight 30-s pulses and 30-s intervals on ice to shear cross-linked DNA. After further centrifugation at 13,000 rpm for 10 min at 4°C, the supernatant was collected, and 10 μL was used as an input sample. Chromatin was added with protein G magnetic beads plus anti-MeCP2 antibody (1:20, Abcam, Cat No: ab2828, RRID:AB_2143853), anti-HDAC1 antibody (1:20, Abcam, Cat No: ab19845, RRID:AB_470299), or anti-HDAC2 antibody (1:20, Cell Signaling Technology, Cat No: #2540, RRID:AB_2116822), and incubated at 4°C overnight. Then protein G magnetic beads were collected and washed sequentially with low-salt buffer, high-salt buffer, LiCl buffer, and Tris–EDTA buffer. Protein/DNA complexes were eluted following the breaking of cross-links and reversed to free DNA. DNA fragments were identified using PCR with the appropriate primers. For all primers, please refer to Supplementary Table 4.

### Co-immunoprecipitation Assay

Co-immunoprecipitation was carried out as described previously (Sharma et al., 2018). L3–L5 DRGs from wild-type mice were collected in lysis buffer containing 50 mmol Tris (pH 8.0), 150 mmol NaCl, 0.1% SDS, 1.0% NP-40, protease inhibitor cocktail, and then homogenized with glass tissue grinders. After centrifugation, the supernatant was collected and then pre-incubated with 50 μL protein A/G mouse agarose (BBI Life Sciences, Shanghai, China; Cat No: 9012-36-6). After overnight incubation, 400 μg protein was incubated with rabbit anti-MeCP2 antibody (Abcam; Cat No: ab2828, RRID:AB_2143853) serum plus Protein A/G mouse agarose and mixed for 4 h at 4°C. After the mixture was washed 4 times with lysis buffer, proteins were eluted by boiling in loading buffer, then separated by SDS-PAGE (Genshare Biological, Cat No: JC-PE022), and finally transferred to a polyvinylidene difluoride membrane (Merck Millipore, Burlington, MSA, United States; Cat No: ISEQ00010) for western blotting. HDACs were detected as described above.

### Cell Culture and Transfection

293T cells were purchased from American type culture collection (ATCC, Manassas). Cells were cultured in DMEM medium (Corning, Cat No: 10-013-CVR) with 10% FBS (Ausbian, Cat No: VS500T). 5% CO2 incubator at 37 °C with water-saturated atmosphere, and 12–12 h light/dark cycle environment was used for cell culture. Cells were transfected with MeCP2-OE or the corresponding negative control (OE-NC) *via* X-tremeGENE HP DNA transfection (ROCHE, Cat No: 06366236001).

### Dual Luciferase Assay

As described in previous literatures (Zhang et al., 2018), the oprm1 promoter region (1316bp) was amplified using standard PCR protocol. PCR products were directionally inserted into the pGL3-Basic luciferase vector, and the DNA sequences were confirmed by direct sequencing. HEK293 cells (3 × 10^4^/well) were seeded and co-transfected MeCP2-OE or the corresponding negative control (OE-NC) with pGL3-Oprm1. Cells transiently transfected with the luciferase constructs were harvested at 48 h. The activities of firefly and Renilla luciferase in cell lysates were measured by a dual-luciferase reporter assay system (Promega, E1910) according to the manufacturer’s instructions. The firefly luciferase to renilla luciferase ratios were determined and defined as the relative luciferase activity. All experiments were performed in triplicate.

### Data Analysis

Both data analyses and chart production were performed using GraphPad Prism 7.00 and ImageJ 2.0. The results are expressed as mean ± standard error of the mean (SEM). Statistical analyses for 2 sets of data were performed using two-tailed unpaired Student’s *t-*test. A one-way or 2-way analysis of variance (ANOVA) with *post hoc* Tukey testing was used to analyze data from multiple groups. A statistical test result was considered significant when *p* < 0.05. Data was statistically analyzed in blinded studies. To estimate the group size required for the behavioral experiments, a pilot study was conducted to measure the PWL in 6 mice 7 days after surgery. The standard deviation was 1.62 in the sham (S_1_) group and 2.22 in the CCI (S_2_) group. The deviation (δ) between the two groups was 3.12 s. With α = 0.05 (Z_α/2_ = 1.96), two-tailed test and power of 80% (Z_β_ = 0.842), following the formula below, we calculated the need for 12.2 (≈12) mice per group. The sample sizes in the molecular experiments are based on previous reports and pilot studies in this field (Li et al., 2020; Ma et al., 2021).


(1) N=[(Zα/2+Zβ)⁢S12+S222/δ]2×4

## Results

### Expression Changes of Mu Opioid Receptor and Methyl-CpG Binding Protein 2 in Dorsal Root Ganglion After Peripheral Nerve Injury

First, we examined the expression of MOR and MeCP2 after peripheral nerve injury in the DRG. The level of MeCP2 protein increased significantly while MOR decreased in the injured DRG from day 5 after unilateral chronic constrictive injury surgery. As expected, sham surgery did not change the basal expression of either MOR or MeCP2 proteins during the observation period (Figure 1A). Correlating with the changes in protein level, we also detected a higher level of MeCP2 mRNA and a lower level of MOR mRNA in the CCI group than those in the sham group on day 7 post-CCI (Figure 1B). Immunostaining further confirmed that the MOR protein was dramatically decreased in the ipsilateral DRG, but not in the sham DRG 7 days after CCI (Figure 1C). Additionally, by using triple labeling for MeCP2 or MOR, neuron (NeuN, a specific neuronal marker) or glutamine synthetase (GS, a marker for satellite glial cells), and DAPI (a marker for cellular nuclei), we found that MOR was predominantly located in neuronal cytoplasm (Figure 1Di), and undetected in the nuclei of GS-labeled DRG cells (Figure 1Dii), while MeCP2 was mostly co-expressed with neurons in the nuclei (Figure 1Diii) and a smaller minority of MeCP2 localized in the nuclei of GS-labeled DRG cells (Figure 1Div). We also analyzed the expression of MOR and MeCP2 on the injured side of L3–5 spinal cord 7 days after surgery. Unexpectedly, only protein and mRNA of MeCP2 showed overt increases, while MOR protein showed a decreasing tendency compared with that in the sham group (Figure 1E–G). Immunostaining also showed the reduced intensity of MOR signal in the ipsilateral spinal dorsal horn, but not in the sham dorsal horn (Figure 1H). Our findings indicate the reduction of MOR and increased expression of MeCP2 in the injured DRG and the ipsilateral side of the spinal cord.

### Methylation Status of the *Oprm1* Gene Promoter After Nerve Injury

**FIGURE 3:**
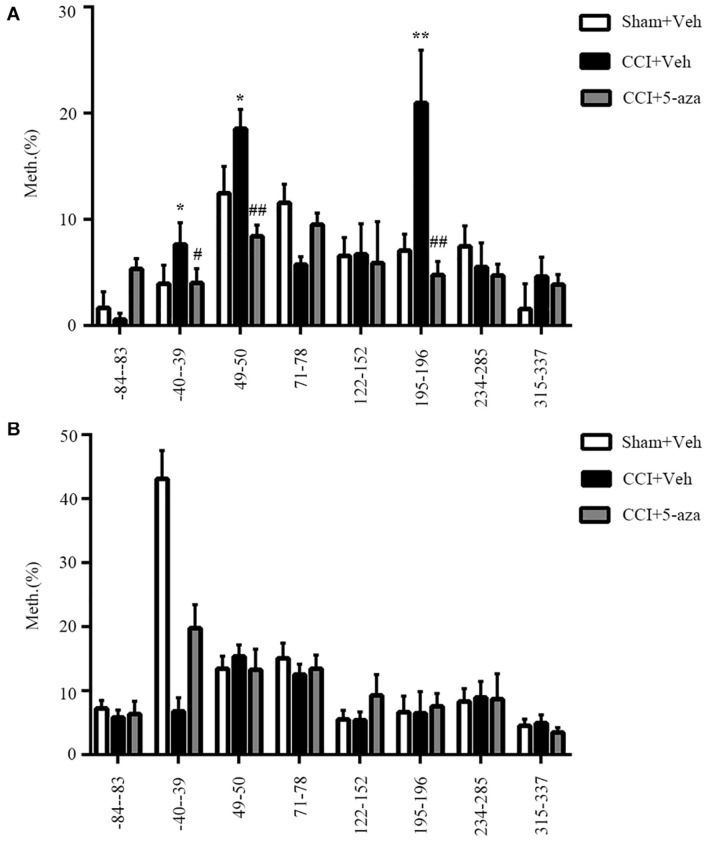
Methylation status of the *Oprm1* gene proximal promoter after nerve injury. **(A,B)** Methylation of the eight regions in the 5′–flanking regions of the *Oprm1* gene (from –85 to +634 bp) in the injured DRG **(A)** and the ipsilateral spinal cord **(B)**. *n* = 3 biological repeats (18 mice)/group in DRG, *n* = 3 biological repeats (3 mice)/group in the spinal cord. One-way ANOVA followed by *post hoc* Tukey test. F_group_ (2, 6) = 15.47, 4.506, 21.62, 16.11, 0.06245, 23.99, 1.814, and 2.291, respectively, for the 8 sites in the DRG. F_group_ (2, 6) = 0.6497, 81.25, 0.6808, 1.156, 3.006, 0.1352, 0.03783, and 1.614, respectively, for the 8 sites in the SC. *p < 0.05, **p < 0.01 versus the sham plus vehicle group; ^#^*p* < 0.05, ^##^*p* < 0.01 versus the CCI plus vehicle group.

Our prior work showed that a methylation inhibitor rescued the analgesia of morphine and that methylation of the *Oprm1* gene promoter increased after nerve injury (Zhou et al., 2014). Therefore, we conducted pyrosequencing to analyze the *Oprm1* gene promoter’s methylation status. We divided the 17 CpG sites in the 5′-flanking regions (from −85 to +634 bp) of the *Oprm1* gene into 8 regions (Table 1) from three groups of animals: Sham plus vehicle group, CCI plus vehicle group, and CCI plus 5-aza-2′-deoxycytidine (5-aza-dC) group. We checked both the ipsilateral DRG and the spinal cord 7 days after surgery. Our work revealed that the methylation status of three of these eight regions (−40 to −39, 49 to 50, and 195 to 196) was higher in the CCI group, while these three regions were demethylated in the CCI plus 5-aza-dC group (Figure 3A). However, there were no obvious hypermethylation changes in the region of the *Oprm1* gene promoter in the ipsilateral spinal cord after nerve injury, with or without 5-aza-dC treatment (Figure 3B). The eight sites are shown in Table 1 (the sites detected here are marked with superscript numbers 1–8 in the rectangular frame).

**TABLE 1:** Eight regions of the 17 CpG islands in the *Oprm1* gene promoter sites.

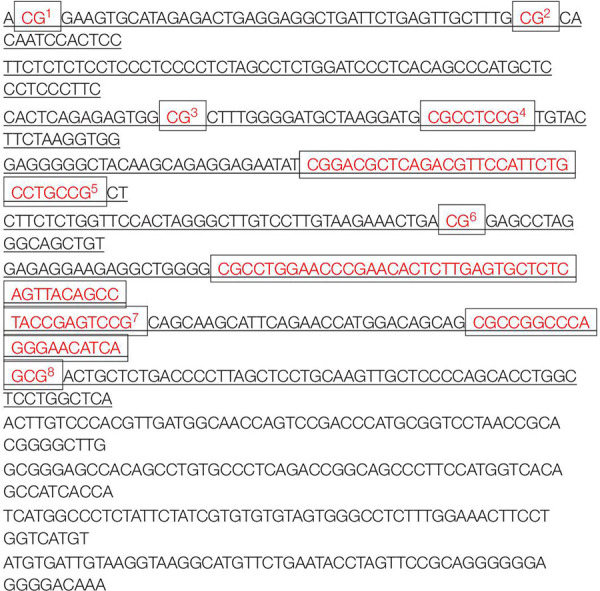

*In *Oprm1* gene promoter regions (from −85 to +634 bp), CpG islands are divided into eight sites, which are marked with superscript numbers 1–8 in the rectangular frame in red font.*

### Methyl-CpG Binding Protein 2 Regulates Mu Opioid Receptor Expression in the Injured Ipsilateral Dorsal Root Ganglion

**FIGURE 4:**
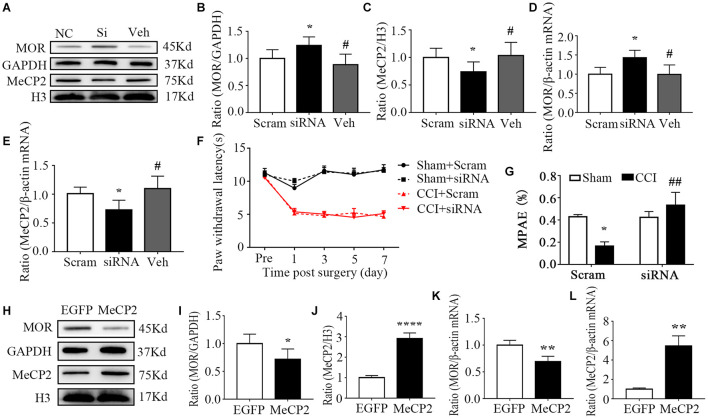
MeCP2 regulated MOR expression in the injured ipsilateral DRG. **(A–G)** The day of surgery was set as day 0. MeCP2 siRNA (100 pmol/10 μL) was intrathecally infused 30 min before surgery for 4 consecutive days. WB and qRT–PCR were undertaken to analyze the expression of MOR and MeCP2 on day 7. PWL was measured 30 min after morphine injection (1.5 mg/kg, subcutaneous infusion, s.c.) on day 7. **(A–C)** Levels of MOR and MeCP2 proteins in the ipsilateral L3–5 DRGs on day 7 after CCI surgery in mice pre-treated with MeCP2 siRNA, scramble RNA or vehicle intrathecally for 4 consecutive days. *n* = 6 biological repeats (18 mice)/group. One-way ANOVA followed by *post hoc* Tukey test. F_group_ (2, 15) = 6.717 for MOR, F_group_ (2, 15) = 3.991 for MeCP2. *p < 0.05, versus the corresponding CCI plus scramble siRNA group; ^#^*p* < 0.05, versus CCI plus MeCP2 siRNA. **(D,E)** Levels of *Oprm1* mRNA, *Mecp2* mRNA in the ipsilateral L3–5 DRGs on day 7 after intrathecal infusion of MeCP2 siRNA (siRNA), control scrambled siRNA (Scram) or vehicle. *n* = 4 biological repeats (8 mice)/group. One-way ANOVA followed by *post hoc* Tukey test. F_group_ (2, 9) = 5.714 for MOR, F_group_ (2, 9) = 5.099 for MeCP2. *p < 0.05, versus the corresponding CCI plus scramble siRNA group. **(F)** Effect of i.t. MeCP2 siRNA or scramble siRNA on the paw withdrawal latencies (PWL) to heat stimuli on the ipsilateral side at the different days after CCI or sham surgery. *n* = 12 mice/group. Two-way RM ANOVA followed by *post hoc* Tukey test. F_group_ (3, 165) = 233.8. **(G)** MPAE showed that the decreased analgesic effect of morphine was rescued through the treatment of MeCP2 siRNA compared with that in the scramble siRNA group on day 7 in neuropathic pain mice. *n* = 12 mice/group. Two-way ANOVA followed by *post hoc* Tukey test. F_group_ (1, 44) = 1.181. *p < 0.05, versus the sham plus scramble siRNA group; ^##^*p* < 0.01, versus the CCI plus scramble siRNA group. **(H–L)** AAV5–MeCP2 or negative control AAV5–EGFP (5*10^^10^VG, i.t.) was used on day 0. WB and qRT–PCR were undertaken to analyze the expression of MOR and MeCP2 on day 36. The levels of MOR protein, MeCP2 protein **(H–J),** *Oprm1* mRNA, and *Mecp2* mRNA **(K,L)** in the L3-5 DRG 36 days after the intrathecal infusion of AAV-EGFP or AAV-MeCP2. *n* = 6 biological repeats (18 mice)/group in panels **(H–J)**. *p < 0.05, ****p < 0.0001, versus the negative control EGFP group by two-tailed unpaired Student’s *t*-test; *n* = 4 biological repeats (8 mice)/group in panels **(K,L)**. **p < 0.01, versus the negative control EGFP group by two-tailed unpaired Student’s *t*-test.

Considering that the methylation of CpG islands was evident in the DRG, we further focused on this area in our subsequent experiments. First, to ascertain whether blocking the increase in DRG MeCP2 could rescue MOR expression, MeCP2-specific short interfering RNA (MeCP2-siRNA, 100 pmol/10 μL, intrathecal infusion, i.t.) was used to knockdown MeCP2 *via* intrathecal infusion on day 0. Negative scramble siRNA was used in the control group. The analgesic effect of morphine was confirmed on day 7 *via* subcutaneous infusion with a dose of 1.5 mg per kg of body weight. As expected, intrathecal infusion of MeCP2 siRNA markedly increased the levels of both protein and mRNA of MOR, compared with the injection of scramble siRNA (Figures 4A,B,D). Meanwhile, the increased expression of MeCP2 in the mouse DRG was absent after the intrathecal infusion of MeCP2 siRNA (not scramble siRNA) at both transcription and translation levels on day 7 post-CCI (Figures 4A,C,E). Consistent with previous studies, CCI led to pain allodynia, but neither siRNA altered paw response to heat on the ipsilateral side of the CCI or sham group (Figure 4F). In addition, morphine analgesia was compromised 7 days after CCI surgery compared with that in the mice of the sham group; however, the injection of mice with MeCP2 siRNA, but not scramble siRNA, prevented this reduction of the analgesic effect of morphine, which was quantified by MPAE (Figure 4G). We further overexpressed MeCP2 in the DRG of normal mice, mimicking the process during neuropathic pain. AAV5 was injected intrathecally to express full-length MeCP2 (5 × 10^10VG, i.t.) into the subarachnoid space of L5 and L6 segments of the spinal cord. As expected, the overexpression of MeCP2 decreased the expression of MOR protein on day 36 post injection (Figures 4H–J). Simultaneously, inducing the expression of MeCP2 also inhibited MOR transcription (Figures 4K,L). From there findings, we concluded that MeCP2 could serve as a key regulator in reducing MOR in DRG after nerve injury.

### HDAC1 Is Involved in the Regulation of Mu Opioid Receptor in the *Oprm1* Promoter Region in the Dorsal Root Ganglion After Nerve Injury

As transcriptional regulators, HDACs are involved in regulating the acetylation of histones and can be recruited by MeCP2 to facilitate transcriptional repression (Nan et al., 1998; Haberland et al., 2009; Beumer and Tawbi, 2010). However, it was unclear whether the same situation would occur in the regulation of MOR expression and which HDAC(s) is involved in this process. We first measured the expressions of HDAC1, –2, –4, and 5, which were reported to be abundant in the nervous system (de Ruijter et al., 2003). Significant increases in the levels of HDAC1, –2, and 5 proteins were observed 7 days after nerve injury, whereas HDAC4 remained unchanged in the ipsilateral DRG (Figure 2A). Similar results were verified regarding the mRNA levels of HDACs on day 7 post-CCI (Figure 2B). Immunostaining further confirmed that the HDAC1, –2 and 5 proteins were dramatically increased in the ipsilateral DRG 7 days after nerve injury (Figure 2C). Interestingly, we observed overt nuclear translocation of HDAC5 after nerve injury (Figure 2Civ). Additionally, immunofluorescence images revealed that HDAC2 was predominantly localized in the nuclei of neurons (Figure 2Dii), whereas other HDACs localized in both nuclei and cytoplasm of DRG neurons in naïve mice (Figures 2Di,iii,iv). Furthermore, using a co-IP assay, we found that MeCP2 antibody could immunoprecipitate HDAC1 and HDAC2, but not HDAC4 or HDAC5, suggesting a specific interaction of MeCP2 with HDAC1 and HDAC 2 in the DRG (Figure 2E). We next performed ChIP assay and revealed that MeCP2 directly bound to sites of *Oprm1* promoter regions (21 to 112, 100 to 234, 206 to 343, and 398 to 511 bp) as the immunoprecipitation of this region (regions from −85 to +634 bp) with MeCP2 antibody showed (Figures 2F,G). Consistently, the dual luciferase assay confirmed the activity of that MeCP2 inhibited the activity of Oprm1 promoter by about 29% (Figure 2H). We further examined the acetylation level of histone H3, as well as HDAC1 and HDAC2 levels, in the promoter region of MOR in both CCI and sham groups. As presented in Figure 2I, HDAC1 but not HDAC2 was detected in this region. The binding activities of HDAC1 in three of the four regions (21 to 112, 100 to 234, and 398 to 511 bp) increased by 1.26-, 1.31-, and 1.21-fold, respectively, in the injured DRG on day 7 after CCI, compared with the levels after sham surgery (Figures 2I,J), while acH3 decreased in the CCI group in these three regions, which was consistent with our expectations (Figures 2I,K).

### Inhibiting Histone Deacetylase Activity Increases Morphine Analgesia and Mu Opioid Receptor Expression in the Injured Ipsilateral Dorsal Root Ganglion

**FIGURE 5:**
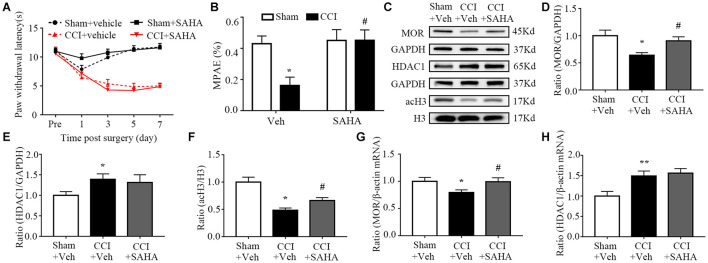
SAHA increased the analgesic effects of morphine and MOR expression in the ipsilateral DRG in neuropathic pain mice. SAHA (50 mg/kg, i.p.) was used 30 min before surgery daily for 7 consecutive days. The day of surgery was set as day 0. PWL was measured 30 min after treatment with morphine (1.5 mg/kg, s.c.) on day 7. WB and qRT–PCR were used to measure protein expression on day 7. **(A)** Effects of i.p. SAHA or vehicle on paw withdrawal latencies (PWL) to heat stimuli on the ipsilateral sides at the different days. Two-way RM ANOVA followed by *post hoc* Tukey test. F_group_ (3, 165) = 124.4. **(B)** The morphine MPAEs measured 30 min after subcutaneous injection of 1.5 mg/kg morphine after SAHA or vehicle treatment from CCI and or sham surgery on day 7. *n* = 12 mice/group. Two-way ANOVA followed by *post hoc* Tukey test. F_group_ (1, 44) = 4.485. *p < 0.05, versus the sham plus vehicle group, ^#^*p* < 0.05, versus the CCI plus vehicle group. **(C–F)** Expression of MOR, HDAC1 and acH3 proteins in the ipsilateral L3–5 DRGs after SAHA or vehicle treatment from the CCI and or sham group. *n* = 6 biological repeats (18 mice)/group. One-way ANOVA followed by *post hoc* Tukey test. F_group_ (2, 15) = 5.593 for MOR, F_group_ (1, 15) = 2.056 for HDAC1, F_group_ (2, 15) = 16.3 for acH3. *p < 0.05, versus the sham plus vehicle group; ^#^*p* < 0.05, versus the CCI plus vehicle group. **(G,H)** Levels of MOR and HDAC1 mRNAs in the ipsilateral L3–5 DRGs on day 7 after i.p. SAHA or vehicle treatment from CCI and or sham mice. *n* = 7 biological repeats (14 mice)/group. One-way ANOVA followed by *post hoc* Tukey test. F_group_ (2, 18) = 3.162 for MOR, F_group_ (2, 18) = 7.065 for HDAC1. *p < 0.05, **p < 0.01, versus the sham plus vehicle group; ^#^*p* < 0.05, versus the CCI plus vehicle group.

Next, we determined whether HDAC blockade could alter the expression of MOR and the analgesic effects of morphine. We corroborated these findings by intraperitoneally administering of SAHA (50 mg/kg, i.p.), a pan-inhibitor of HDACs Neither SAHA nor vehicle altered paw responses to heat stimuli on the ipsilateral side of CCI mice and sham mice (Figure 5A). Like in MeCP2 siRNA treated mice, SAHA inhibition was associated with a greater analgesic effect of morphine than in the vehicle group on the ipsilateral side of CCI mice (Figure 5B). Additionally, both protein and mRNA levels of MOR expression were reduced in injured DRG on day 7 post-CCI, which were rescued by SAHA injection (Figures 5C,D,G). The increased expression of HDAC1 was also observed, which was not affected by the infusion of SAHA, as SAHA could only inhibit its activity rather than its expression (Figures 5C,E,H). Concurrently, we detected a global decrease of acetylation of histone H3 in the injured DRG, while SAHA prevented this reduction (Figure 5F). Our results indicate that MOR downregulation could be rescued by SAHA, leading to an enhanced analgesic effect of morphine in neuropathic pain mice.

## Discussion

In the present study, we demonstrated that peripheral nerve injury led to increases of MeCP2 and HDAC1 expression in injured DRG. Increased MeCP2 and HDAC1 accumulated around *Oprm1* gene promoter sites, contributing to a decrease of acetylation of H3 in *Oprm1* gene promoter region and being associated with the downregulation of MOR expression in the injured DRG. The study uncovers a previously unknown facet of MOR reduction in the peripheral nervous system at the molecular level.

Methylation of the Oprm1 gene promoter is one mechanism driving the downregulation of Oprm1 gene expression in the DRG ipsilateral to the site of injury. In fact, distinct molecular mechanisms, including DNA methylation, histone methylation, and microRNA as well as post-transcriptional mechanisms, such as RNA N6-methyladenosine modification (m6A), also contribute to this downregulation. DNA methyltransferases 3a (DNMT3a)-triggered DNA methylation is involved in nerve trauma-induced downregulation of MOR in the DRG, and miR-143 directly interacts with this transcription factor to influence MOR expression (Sun et al., 2017; Xu et al., 2017). The level of another transcription factor, Octamer transcription factor 1, was also increased during peripheral nerve trauma, which activates *Dnmt3a* gene transcriptional activity and elevates *Dnmt3a* mRNA protein (Yuan et al., 2019). The *Oprm1* gene is transcriptionally activated by the transcription factor cAMP response element-binding protein (CREB), while MBD1, which is elevated in injured DRG neurons, blocks the access of CREB to the promoter of the *Oprm1* gene and downregulates the expression of *Oprm1* mRNA and MOR protein (Mo et al., 2018). G9a, a histone methyltransferase, encoded by the *Ehmt2* gene, produces histone H3 dimethylation at Lys9. Nerve injury was shown to increase G9a protein, contributing to increased methylation of histone3 in *Oprm1* gene promoter sites, and caused chromatin compaction as well as the downregulation of *Oprm1* in nerve trauma-related DRG (Liang et al., 2016; Zhang et al., 2016). Additionally, a recent study reported that peripheral nerve injury downregulates DRG-specifically enriched lncRNA (DS-lncRNA) in injured DRG. Rescuing this downregulation blocked nerved injury-induced increases in *Ehmt2* mRNA and its coding protein, G9a, and promoted *Oprm1*, *Oprk1*, *Oprd1*, and *Kcna2* mRNAs in injured DRG (Pan et al., 2021). Gene expression is also regulated by RNA modifications. The level of fat-mass and obesity associated proteins (FTO), an m6A eraser protein, was found to time-dependently increase in the ipsilateral L5 DRG after spinal nerve ligation. Increased FTO may maintain the nerve trauma-induced increase in G9a expression and consequent downregulation of G9a-determined MOR in nerve trauma-related DRG neurons (Li et al., 2020). Taking these findings together, DNMT3a, G9a, DS-lncRNA and FTO are likely potential targets for adjunctive use with opioids in the management of neuropathic pain.

The transcription factor, MeCP2 is expressed widely in the nervous system. MeCP2 mutation leads to classical Rett syndrome, and pain hypoalgesia was reported in Rett syndrome patients. Recently, increased MeCP2 expression has also been observed in different pain models (Tochiki et al., 2012; Suzuki et al., 2016; Xie et al., 2018). For instance, in a model of pelvic inflammation, analysis of DRG sections revealed that the number of MeCP2-positive neurons was increased in mice with colonic inflammation (Xie et al., 2018). The expression of transient receptor potential vanilloid 1 was also found to contribute to tongue heat sensitivity and hypersensitivity, and was regulated by MeCP2 signaling in trigeminal ganglion neurons innervating the tongue (Suzuki et al., 2016). MeCP2 was shown to be abundantly expressed in neuronal as well as glial cells. Inflammation- related genes, such as *Il6*, *Tnf*, *Cxcl2*, and *Cxcl3*, in microglia and macrophages, were also found to be regulated by MeCP2 in response to inflammatory stimuli (Cronk et al., 2015). In addition, a recent study revealed that pain symptoms could be transmitted across generations, and Tao et al. identified an essential role of MeCP2, which showed increased expression in primary somatosensory cortical (S1) glutamate neurons in offspring mice, in this process (Tao et al., 2020). These results collectively suggest the importance of MeCP2 as a key regulator in pain epigenetic regulations. In the present study, we found that the *Mecp2* gene in DRG can be activated at the transcriptional level in response to peripheral nerve injury. The level of *Mecp2* mRNA and the protein that it encodes increased in the injured DRG, but not in the sham group, which was consistent with some prior studies (Wang et al., 2011; Tochiki et al., 2012; Manners et al., 2016). Reducing the CCI-induced increase in DRG *via* the intrathecal injection of MeCP2 siRNA could rescue MOR expression, and its overexpression in wild type mice lowered MOR expression, further confirming the regulatory effects of MeCP2. However, other studies presented the opposite results. For example, one study showed that the development of neuropathic pain was attenuated in MeCP2 transgenic mice, suggesting an analgesic role of MeCP2 (Zhang et al., 2015), while we found no obvious anti-nociceptive effect of treatment with MeCP2 siRNA (Figure 4F). This discrepancy might be related to the different animals used, as Zhang et al. used MeCP2 transgenic mice while we only verified our findings *via* the intrathecal infusion of MeCP2 siRNA. These results indicate the complex roles of MeCP2 in the pain pathways of the peripheral and central nervous system, so more works on this issue is needed for clarification.

A previous study proposed that HDACs are involved in pain formation. For example, Kv1.2 was shown to be inhibited by HDAC2 in injured DRG (Li et al., 2019a), influencing the excitability of neurons, which contributed to the induction and development of neuropathic pain. HDAC1 was also shown to be associated with c-Jun in the nuclei of spinal dorsal horn astrocytes expressing c-Jun N-terminal kinase, triggering the activation of other inflammatory factors (Sanna and Galeotti, 2018). Moreover, in a complete Freund’s adjuvant inflammation pain model, intrathecal injection of an HDAC inhibitor increased K^+^-Cl^–^ co-transporter expression by restoring the spinal inhibitory synaptic transmission (Lin et al., 2017). In addition, treatment with histone deacetylase inhibitors relieved the pain by normalizing glutamate decarboxylase 65 in the rat brainstem nucleus raphe magnus (Zhang et al., 2011). Nerve injury can also induce acetylation of histones H3 and H4, and the increased transcription of inflammatory molecules, such as macrophage inflammatory protein-2 or C-X-C chemokine receptor type 2, which intensifies or prolongs pain (Gao and Ji, 2010; Kiguchi et al., 2012; Sun et al., 2013). Thus, we should pay close attention to the complex implications of HDACs from both research and clinical perspectives.

Interestingly, we noted that nerve injury promoted the translocation of HDAC5 into the nucleus (Figure 2Civ). In fact, Gu et al. (2021) reported that the spinal administration of endothelin-1 promoted the nuclear efflux of HDAC5 in the spinal cord and attenuated the nociception induced by partial sciatic nerve ligation surgery. However, the role of the nuclear translocation of HDAC5 in injured DRG remains unknown. We will examine these mechanisms in future work. Furthermore, there was a trend for the downregulation of MOR in the spinal cord, although this did not reach significance (Figures 1E–H). One reason for this could be that a substantial proportion of MOR in the spinal cord was transported from DRG somas to their central terminals, as Sun et al. found that ablating MOR in DRG markedly reduced MOR expression of MOR in the dorsal horn of the spinal cord (Sun et al., 2019, 2020). We conjectured that this explained why MOR protein levels decreased while mRNA levels remained stable in the ipsilateral spinal cord.

Some limitations of our study should be mentioned. Neuropathic pain is defined by the International Association for the Study of Pain as pain caused by damage or disease affecting the somatosensory nervous system (Finnerup et al., 2016). Approximately 15–25% of chronic pain is neuropathic, with the most common conditions including diabetic neuropathy, postherpetic neuralgia, and radiculopathy (Cohen and Mao, 2014). In fact, opioid levels in the peripheral nervous system were found to be decreased in both experimental diabetes mice and approximately half of patients with painful diabetic peripheral neuropathy (Deshpande et al., 2021). In addition, morphine was found to inhibit herpetic pain more effectively than postherpetic pain induced by herpetic virus inoculation for the specific downregulation of MOR in primary sensory neurons in mice (Takasaki et al., 2006). Our study focused on an animal model of neuropathic pain resulting from CCI surgery, a form of peripheral mononeuropathy. However, the decrease of MOR in neuropathic pain caused by other triggers, such as damage induced by metabolic dysfunction in diabetes or viral infection remains uncharacterized. Moreover, chronic pain and high-impact chronic pain are more prevalent in women (Dahlhamer et al., 2018), while Rett syndrome, which results from mutations in MeCP2, is almost exclusively found in females as the gene is X-linked (Sandweiss et al., 2020). It is also becoming increasingly clear that there are huge differences in the molecular, cellular and system-level mechanisms of chronic pain processing in male and female rodents and humans (Sorge et al., 2015; Luo et al., 2019). The mechanisms by which MeCP2 exerts effects on MOR expression in male mice may differ from those in female mice, which requires more research. Another limitation we ‘d like to mention is that, the general understanding in the field is that the antinociceptive actions of opioid analgesics is derived from receptors expressed in the spinal cord and central nervous system (CNS). Intrathecal administration of siRNA, viral vectors and epigenetic modifying agents would also target these sites. Furthermore, both MeCP2 and HDACs are widely expressed in CNS cells. As a result, we couldn’t rule out their influences on these sites in this paper.

In conclusion, after peripheral nerve injury, the methylation of CpG islands in the promoter region of the *Oprm1* gene was found to be increased. This increase recruited MeCP2 protein, which directly bound to HDAC1, reducing histone acetylation, and restrained the transcription of *Oprm1*. As a result, the expression of MOR was reduced. Inhibiting MeCP2/HDAC1 may restore MOR expression to induce a better analgesic profile of morphine.

## Data Availability Statement

The raw data supporting the conclusion of this article will be made available by the authors, without undue reservation.

## Ethics Statement

The animal study was reviewed and approved by Animal Care and Use Committee of Zhejiang University.

## Author Contributions

NS, LY, and MY participated in the study design. NS, YG, JR, LW, JH, YL, and CX conducted the experiments. YW and YL performed the data analysis. NS, LM, JR, and DG wrote or contributed to the manuscript discussion. All authors contributed to the article and approved the submitted version.

## Conflict of Interest

The authors declare that the research was conducted in the absence of any commercial or financial relationships that could be construed as a potential conflict of interest.

## Publisher’s Note

All claims expressed in this article are solely those of the authors and do not necessarily represent those of their affiliated organizations, or those of the publisher, the editors and the reviewers. Any product that may be evaluated in this article, or claim that may be made by its manufacturer, is not guaranteed or endorsed by the publisher.
