# Supplementary material for: MeCP2 Epigenetic Silencing of Oprm1 Gene in Primary Sensory Neurons Under Neuropathic Pain Conditions
Source: Front Neurosci. 2021 Nov 5;15:743207. doi: 10.3389/fnins.2021.743207 (PMC8602696; doi:10.3389/fnins.2021.743207)
Supplement: Supplementary file 1 [file Data_Sheet_1.docx]

Ⅰ. Table S1. Primers and sequences used in qRT-PCR.

| Gene Name | Primers | Sequences 5’to 3’ |
| --- | --- | --- |
| mHdac1 | Forward | ACGGCATTGACGACGAATCCTATG |
|  | Reverse | CTGAGCCACACTGTAAGACCACTG |
| mHdac2 | Forward | TGATGGAGATGTACCAGCCTAGCG |
|  | Reverse | ACCGAGCATCAGCAATGGCAAG |
| mHdac4 | Forward | CCAAGCCAAGGTTCACCACAGG |
|  | Reverse | GCGTCCACGGATGCACTCAC |
| mHdac5 | Forward | AGCACCGAGGTAAAGCTGAG |
|  | Reverse | GAACTCTGGTCCAAAGAAGCG |
| mMeCP2 | Forward | CAGAAACATCAGAAAGCTCAGG |
|  | Reverse | GGCCAGACTTCCTTTGTTTAAG |
| mOprm1 | Forward | GGATCGAACTAACCACCAGCCAAC |
|  | Reverse | GGTTCCTCATTCCTCTGTCCATGC |
| mβ-actin | Forward | CTACCTCATGAAGATCCTGACC |
|  | Reverse | CACAGCTTCTCTTTGATGTCAC |

qRT-PCR: quantitative real-time–PCR.

Ⅱ. Table S2. The primers and the sequences used in pyrosequencing.

| **Primer name** | Sequences 5’to 3’ | **5'modification** |
| --- | --- | --- |
| Oprm-1F | ATGTATGGTTGTTATAAAGAAATTTAGAGT | 5'-Biotin |
| Oprm-1R | TCAAAATCAACCTCCTCAATCTCTAT |  |
| Oprm-1S | CTCCTCAATCTCTATACAC |  |
| Oprm-2F | AGATTGAGGAGGTTGATTTTGAGTTG |  |
| Oprm-2R | TACTTATAACCCCCTCCCACCTTAAA | 5'-Biotin |
| Oprm-2S | GGTTGATTTTGAGTTGT |  |
| Oprm-3F | AGAGATTGAGGAGGTTGATTT | 5'-Biotin |
| Oprm-3R | AACCCCCTCCCACCTTAAA |  |
| Oprm-3S | CATCCTTAACATCCCC |  |
| Oprm-4F | GAGTGGAGTTTTGGGGATGTTAAGGA |  |
| Oprm-4R | ACCCCCTCCCACCTTAAAA | 5'-Biotin |
| Oprm-4S | GGGGATGTTAAGGAT |  |
| Oprm-5F | TGGGAGGGGGTTATAAGTAGA |  |
| Oprm-5R | CTCTCACAACTACCCTAAACTCC | 5'-Biotin |
| Oprm-5S | GGGGTTATAAGTAGAGGA |  |
| Oprm-6F | TAAGGTGGGAGGGGGTTATAAGTAG |  |
| Oprm-6R | ACCTCTTCCTCTCACAACTACC | 5'-Biotin |
| Oprm-6S | GGGTTTGTTTTTGTAAGAAAT |  |
| Oprm-7F | AGTTTAGGGTAGTTGTGAGAG |  |
| Oprm-7R | ACCAAAAACCAAATACTAAAAAACAACTT | 5'-Biotin |
| Oprm-7S | GAGGAAGAGGTTGGG |  |
| Oprm-8F | AGTTTAGGGTAGTTGTGAGAG |  |
| Oprm-8R | ACCAAAAACCAAATACTAAAAAACAACTT | 5'-Biotin |
| Oprm-8S | AGTAAGTATTTAGAATTATGGATAG |  |

F: forward primer; R: reverse primer; S: sequencing primer.

Ⅲ. Table S3. The siRNAs of MeCP2 and related primer duplexes.

| Gene name | Sequences 5’to 3’ |
| --- | --- |
| *Mecp2*-siRNA-1 | Sense 5’-CGCUCUAAAGUAGAAUUGAUU-3’ |
|  | Antisense 5’-AAUCAAUUCUACUUUAGAGCG-3’ |
| *Mecp2*-siRNA-2 | Sense 5’-CCUAAGAAGGAGCACCAUCAU-3' |
|  | Antisense 5’-AUGAUGGUGCUCCUUCUUAGG-3' |
| *Mecp2*-siRNA-3 | Sense 5’-CCCUGGGAAACUUGUUGUCAA-3’ |
|  | Antisense 5’-UUGACAACAAGUUUCCCAGGG-3’ |

Ⅳ. Table S4. The primers and the sequences used in ChIP.

| **Primer name** | Sequences 5’to 3’ |
| --- | --- |
| Oprm-1F | CATGCTCCCTCCCTTCCACT |
| Oprm-1R | GTAGCCCCCTCCCACCTTAG |
| Oprm-2F | GGGGCTACAAGCAGAGGAGA |
| Oprm-2R | CCCAGCCTCTTCCTCTCACA |
| Oprm-3F | AGGGCAGCTGTGAGAGGAAG |
| Oprm-3R | CAGTCGCTGATGTTCCCTGG |
| Oprm-4F | CTTGTCCCACGTTGATGGCA |
| Oprm-4R | ATGGCTGTGACCATGGAAGG |

F: forward primer; R: reverse primer.
